# Intratypic heterologous vaccination of calves can induce an antibody response in presence of maternal antibodies against foot-and-mouth disease virus

**DOI:** 10.1186/1746-6148-10-127

**Published:** 2014-06-06

**Authors:** Aldo Dekker, Phaedra Eblé, Norbert Stockhofe, Gilles Chénard

**Affiliations:** 1 Central Veterinary Institute of Wageningen UR, P.O. Box 65, 8200 AB Lelystad, The Netherlands; 2 Boehringer Ingelheim Animal Health Operations, C.J. van Houtenlaan 36, 1381 CP Weesp, The Netherlands

**Keywords:** Foot-and-mouth disease, Maternal antibodies, FMD, FMDV, Vaccine, Emergency vaccination, Control, Half-life, r1 value, Heterologous vaccination

## Abstract

**Background:**

Maternal antibodies can interfere with foot-and-mouth disease vaccination. In this study we determined whether intratypic heterologous vaccination could help to improve herd immunity.

**Results:**

In unvaccinated calves, a half-life of maternal antibodies of 21 days was determined. At two weeks of age, calves without maternal antibodies showed a good antibody response against both vaccines used in the trial, while in calves with maternal antibodies no antibody response to homologous vaccination (A Turkey 14/98) but a limited antibody response to intratypic heterologous vaccination (A_22_ Iraq) was observed.

**Conclusion:**

Two weeks old calves without maternal antibodies respond well to vaccination, but when emergency vaccination is carried out in a region that uses prophylactic vaccination, using an intratypic heterologous vaccine strain may improve the immunity in calves with maternal antibodies.

## Background

Maternal antibodies against foot-and-mouth disease virus (FMDV) interfere with vaccination [1-3]. This interference is not unique for FMDV but is seen in many diseases and can cause the occurrence of a serious immunity gap in young animals of a population. This immunity gap is due to waning levels of maternal antibodies and poor response to vaccination in the presence of maternal antibodies. In the case of canine distemper, a solution to overcome this immunity gap was the use of measles virus, a related Morbillivirus, for the vaccination of newborn puppies. Vaccination with measles virus could induce active immunity against canine distemper in the presence of maternal antibodies against canine distemper. However, although in vaccination trials a good response was seen, the vaccine was less successful in field conditions [4].

Prophylactic vaccination against FMDV is often practised in endemically infected areas. Also here, the immunity gap in young animals needs special attention. Outbreaks in countries that use prophylactic vaccination are often seen in areas or in age groups with poor immunity against the disease. Emergency vaccination around the observed outbreaks can improve protection against FMDV. However, prophylactic vaccination is usually not applied before the age of 3–4 months [1]. However, in an outbreak situation, emergency vaccination has to start immediately and one cannot wait till the young animals have reached the age that normally would be considered optimal for vaccination. But then maternal antibodies will interfere with the response. Then the use of an intratypic heterologous vaccine in order to overcome the effect of maternal antibodies might be considered.

Such a situation occurred during the FMDV outbreak in Turkish Trace in early 2006. All dams had previously been vaccinated with an FMDV type A IRN/96 (or similar strain). The 2006 FMDV outbreak strain, however, showed a better match with A_22_ Iraq. Therefore A_22_ Iraq antigen was included in the emergency vaccine. Normally, young cattle would be vaccinated after levels of maternal antibodies had sufficiently declined. But here the question was raised whether calves with maternal antibodies against A IRN/96 would respond to the A_22_ Iraq strain used in the emergency vaccine, because this strain was heterologous in respect to the maternal antibodies. Based on the results with measles and canine distemper described above, it was advised to vaccinate all ruminants irrespective of age.

To elucidate whether the given advice was sound, we investigated whether intratypic heterologous vaccination using A_22_ Iraq could successfully induce an antibody response in young calves and especially if born from dams vaccinated against A Turkey 14/98 (that falls within the A IRN/96 lineage).

## Methods

The animal experiments received approval of the animal ethics committee of the Central Veterinary Institute in accordance with the Dutch law ("wet op de dierproeven"). The animals were housed and fed according to the standard operating procedures at the animal facilities of the Central Veterinary Institute, which is similar to the housing on farms in the Netherlands. All cows and calves were conventionally reared full and mixed breed Holstein Frisians. Calves were assigned to 5 groups of 5 calves (Table 1). The first 15 calves (groups 1 to 3) were fed with colostrum obtained from 15 cows that had been used in a vaccine safety trial. To make sure that the birth vaccination interval was similar and vaccination in each group could be performed on the same day, we assigned the first 5 calves born to group 1, the next 5 calves to group 2 and the last 5 calves to group 3. Calves in group 4 and 5 arrived on the same day and were randomly assigned to the groups. The dams provided the colostrum to their own calves, but of a few dams a part of the colostrum was frozen. If a calf died it was replaced by a colostrum deprived one day old calf, which was fed with the colostrum stored in the freezer. The dams that provided colostrum to the calves in groups 1, 2 and 3, had been vaccinated three times with a trivalent (A Turkey 14/98, O Manisa, Asia-1 Shamir) Cedivac-FMD vaccine [5], one time in each trimester of pregnancy. Calves used in groups 4 and 5 were born from non-vaccinated dams. Calves of groups 1, 2, 4 and 5 were vaccinated two to three weeks after birth. The calves in groups 1 and 4 were vaccinated subcutaneously with 2 ml of a monovalent homologous Cedivac-FMD vaccine containing strain A Turkey 14/98 and the calves in groups 2 and 5 were vaccinated similarly with a heterologous monovalent Cedivac-FMD vaccine containing strain A_22_ Iraq (Table 1). The formulation (antigen dose and adjuvant) of both vaccines were similar. Serum samples were collected on a weekly basis up to 6 weeks after vaccination and tested for virus neutralising antibodies against FMDV type A_22_ Iraq, A Turkey 14/98, O_1_ Manisa and Asia 1 Shamir, using BHK cells (performed according tot the OIE manual, http://www.oie.int).

**Table 1:** Experimental design (5 calves in each group)

**Group**	**Maternal antibodies**	**Cedivac-FMD vaccine**
1	Yes	A Turkey 14/98
2	Yes	A_22_ Iraq
3	Yes	None
4	No	A Turkey 14/98
5	No	A_22_ Iraq

Two-fold dilutions of the serum samples were tested starting with undiluted serum. For calculation of the mean titres and for the use in statistical tests we used 0 for the observations with a log_10_ titre of <0.30. To calculate the r1-value [6] between the FMDV strains A Turkey 14/98 and A22 Iraq, we used the geometric mean titres of the serum samples collected at 3 weeks post-vaccination from the calves without maternal antibodies (groups 4 and 5). The relation between neutralising antibody titre in the dams 4 weeks after the last vaccination (approximately 30 weeks of gestation) and the antibody titre in the calves just after colostrum uptake was analysed by least-square regression.

The decrease of maternal antibodies and the effect of the different vaccination strategies was analysed using a linear mixed effects model, because of the repeated sampling of the same calves. In this linear mixed effects model, animal was the random variable, the neutralising antibody titre was the response variable and age, serotype and group the possible explanatory variables. By using the animal as random variable, parallel regression lines per calf can be estimated as not all calves start with the same titre. In this analysis neutralising antibody titres <0.3 were excluded, as we were only interested in the part of the curve that could be measured. Using a forward stepwise method the various explanatory variables were introduced into the model. The best fitting model was selected based on the AIC. All the statistical analyses were performed using R (http://www.r-project.org) version 2.14.2 [7]. For the linear mixed effects model, the lme4 library was used [8].

## Results

### Maternal antibodies

**Figure 1:**
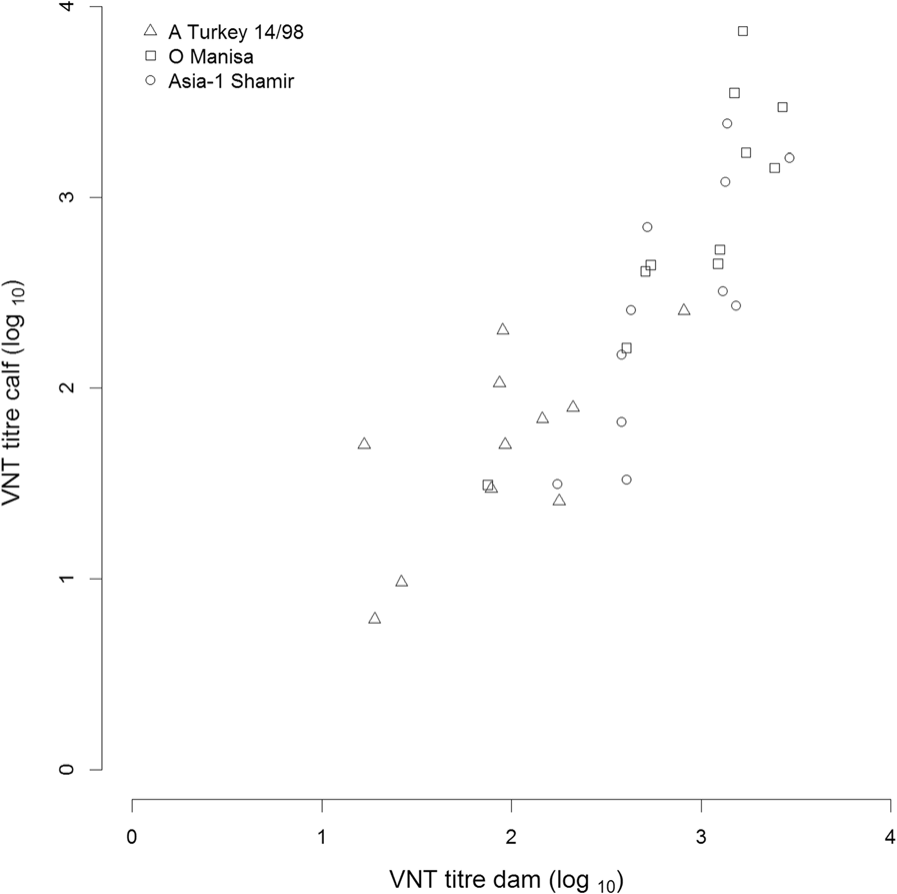
**Relation between neutralising antibody titre (log**_**10**_**) in the dams 2 weeks after the last vaccination (approximately 30 weeks of gestation) and the antibody titre in the calves just after colostrum uptake.**

A total of 4 calves died in the first 14 days after birth, 1 calf in group 2 and 3 calves in group 3; these calves were replaced with other calves that were subsequently fed with colostrum from one of the vaccinated dams. In the 11 calves that survived, the amount of maternal antibodies varied between calves (Figure 1). Although uptake of colostrum is essential for obtaining maternal antibodies, analysis of the neutralising antibody titre observed in the dam and the offspring showed that the magnitude of the maternal antibody titre in healthy calves correlated with the neutralising antibody titre of the dam. The response in the dams and therefore in the calves, varied for the different strains. Statistically there was no significant difference in the slope of the relation between neutralising antibody titre of the dams and their calves. For all three serotypes combined, the slope of the relation between neutralising antibody titre in the dam and the calf was 1.1 and the intercept -0.5 (R-squared 0.72, Figure 1).

### Half-life maternal antibodies

The analysis of the mixed linear effects model of the observed maternal antibody titres of groups 1, 2 and 3 for all 3 FMDV serotypes included in the vaccine showed that the observed maternal antibody titre was explained by the serotype and the age of the calves (Table 2). Although the level of antibodies was significantly different between serotypes (p < < 0.001), no significant interaction between serotype and age was found. This means that no difference in half-life of maternal antibodies was observed between the serotypes. There was neither a significant interaction between group and age which means that there was no difference in half-life values between the three groups of calves with maternal antibodies. The latter shows that neither the vaccination with strain A Turkey 14/98, nor strain A_22_ Iraq had an influence on the decrease of maternal antibodies. An average half-life of 21 days was calculated (95% confidence interval 19–24 days).

**Table 2:** Estimates, standard error and t-value of the fixed effects in the final linear mixed effects model explaining the neutralising antibody titres in the calves with maternal antibodies

	**Estimate**	**Standard error**	**t-value**
Intercept	2.7	0.15	18
Strain O Manisa	Reference		
Strain Asia-1 Shamir	-0.21	0.039	-5.3
Strain A Turkey 14/98	-0.93	0.040	-23
Age	-0.014	0.000083	-17

### Vaccination response in the calves

**Figure 2:**
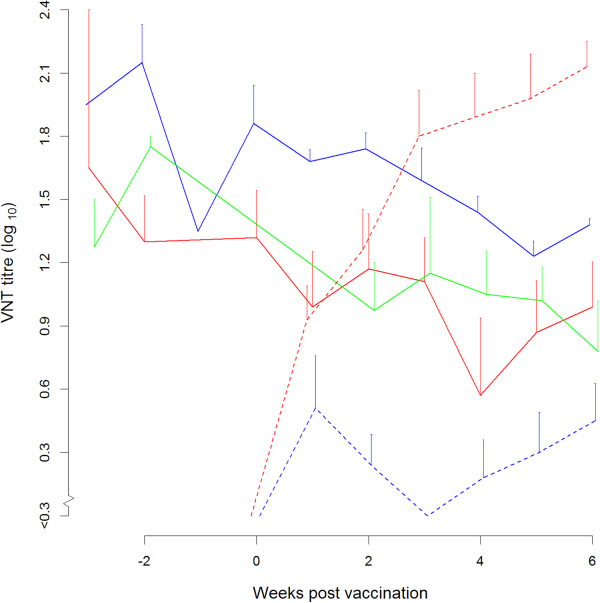
**Average neutralising antibody titres against A Turkey 14/98 observed in serum samples from calves vaccinated with monovalent A Turkey 14/98 vaccine (red) or with A**_**22 **_**Iraq vaccine (blue) and from the non-vaccinated calves with maternal antibodies (green).** The average neutralising antibody titres of the groups with maternal antibodies (group 1, 2 and 3) are shown with a solid line. The average neutralising antibody titres of the calves without maternal antibodies (group 4 and 5) are shown a dashed line. The bars represent the standard error of the mean; to prevent overlapping error-bars the lines are slightly displaced.

**Figure 3:**
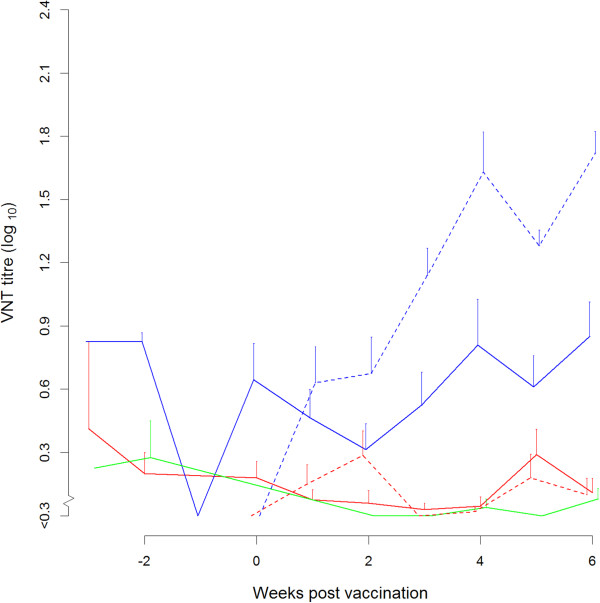
**Average neutralising antibody titres against A**_**22 **_**Iraq observed in serum samples from calves vaccinated with monovalent A Turkey 14/98 vaccine (red) or with A**_**22 **_**Iraq vaccine (blue) and from the non-vaccinated calves with maternal antibodies (green).** The average neutralising antibody titres of the groups with maternal antibodies (group 1, 2 and 3) are shown with a solid line. The average neutralising antibody titres of the calves without maternal antibodies (group 4 and 5) are shown a dashed line. The bars represent the standard error of the mean; to prevent overlapping error-bars the lines are slightly displaced.

The mean interval between birth and vaccination was 2.6, 2.3, 2.9 and 2.9 weeks for groups 1, 2, 4 and 5 respectively (individually ranging from 2.0 to 3.3 weeks). The two-three week old calves without maternal antibodies showed a good homologous antibody response after vaccination (dotted lines, groups 4 and 5 in respectively Figures 2 and 3). The calves without maternal antibodies did not show a cross-neutralising antibody response against the heterologous type A; in all cases heterologous titres at 3 week post vaccination were < 0.3. This resulted in a mean r1-value of less than 0.1 for all serum samples obtained from A Turkey 14/98 vaccinated calves when tested again A_22_ Iraq virus. And a mean r1-value of 0.1 for the all A_22_ Iraq vaccinated calves when tested with A Turkey 14/98 virus.Calves born from vaccinated dams all had maternal antibody titres against A Turkey 14/98 that was included as antigen in the vaccine of the dams (Figure 2). The calves with maternal antibodies against A Turkey 14/98 did not respond to homologous vaccination with A Turkey 14/98. The average neutralising antibody titre against A Turkey 14/98 of group 1 (solid red line, Figure 2) is still decreasing after vaccination of the calves and overlaps with the average neutralising antibody titre of the non-vaccinated calves of group 3 (solid green line, Figure 2).

The calves with maternal antibodies against A Turkey 14/98 (group 2, solid blue line Figure 3) that were vaccinated with the heterologous strain A_22_ Iraq, however, did respond to vaccination; their antibody titres are significantly higher (p = 0.002) than the titres of the calves with maternal antibodies against A Turkey 14/98 that were not vaccinated (group 3, solid green line Figure 3).

## Discussion

While the experiment had originally been started as a vaccine safety trial (the dams had been vaccinated three times during pregnancy), it was decided to extend the trial in order to examine whether FMD vaccination of young calves makes sense in general and in particular, whether an intratypic heterologous vaccine could induce an antibody response in the presence of maternal antibodies. In two-week old calves without maternal antibodies, a good antibody response to both A Turkey 14/98 as well as A22 Iraq (group 4 and 5, the dotted lines in Figures 2 and 3) was observed. Obviously, the immune system of calves is capable of responding to vaccination already at this age. In contrast to calves without maternal antibodies, calves with high levels of maternal antibodies did not respond to vaccination with a homologous vaccine, confirming earlier findings [1,3]. However, a limited antibody response was seen after intratypic heterologous vaccination in such calves. Apparently, intratypic heterologous vaccination can, to some extent, circumvent the inhibiting effect of maternal antibodies. The experimental set-up well reflected a typical field situation. The neutralising antibody titres in the multiply-vaccinated dams were high, but high neutralising antibody titres may also be expected in infected dams, in which case in their calves maternal antibodies can probably be detected for more than 6 months. In the non-vaccinated calves an average half-life of 21 days was estimated, which corresponds very well with earlier findings (22 days [3]).

## Conclusion

This study shows that the immune system of calves is capable of responding to vaccination at 2 weeks of age and that intratypic heterologous FMDV vaccination can circumvent the inhibiting effect of maternal antibodies albeit to a limited level.

## Abbreviations

FMDV: Foot-and-mouth disease virus.

## Competing interests

The authors declare that they have no competing interests.

## Authors' contributions

All authors contributed in the design of the study. GC and NS participated in the coordination of the study. AD was responsible for the analysis of the data. AD drafted the article. NS, PE and GC contributed all to the final version of the manuscript and approved the final manuscript. All authors read and approved the final manuscript.
